# The Relationship Between Obesity and Depression Is Partly Dependent on Metabolic Health Status: A Nationwide Inpatient Sample Database Study

**DOI:** 10.3389/fendo.2022.880230

**Published:** 2022-05-25

**Authors:** Zhixiang Wang, Yiping Cheng, Yuan Li, Junming Han, Zhongshang Yuan, Qihang Li, Fang Zhong, Yafei Wu, Xiude Fan, Tao Bo, Ling Gao

**Affiliations:** ^1^Department of Endocrinology, Shandong Provincial Hospital Affiliated to Shandong First Medical University, Jinan, China; ^2^Shandong Clinical Research Center of Diabetes and Metabolic Diseases, Jinan, China; ^3^Shandong Key Laboratory of Endocrinology and Lipid Metabolism, Jinan, China; ^4^Shandong Prevention and Control Engineering Laboratory of Endocrine and Metabolic Diseases, Jinan, China; ^5^Department of Endocrinology, Shandong Provincial Hospital, Cheeloo College of Medicine, Shandong University, Jinan, China; ^6^Department of Biostatistics, School of Public Health, Cheeloo College of Medicine, Shandong University, Jinan, China; ^7^Central Laboratory, Shandong Provincial Hospital Affiliated to Shandong First Medical University, Jinan, China

**Keywords:** metabolic obesity phenotype, depression, sex difference, metabolic abnormalities, age difference, obesity phenotype

## Abstract

**Objective:**

Some studies have demonstrated a bidirectional association between obesity and depression, whereas others have not. This discordance might be due to the metabolic health status. We aimed to determine whether the relationship between obesity and depression is dependent on metabolic health status.

**Methods:**

In total, 9,022,089 participants were enrolled and classified as one of four obesity phenotypes: metabolically healthy nonobesity (MHNO), metabolically unhealthy nonobesity (MUNO), metabolically healthy obesity (MHO), and metabolically unhealthy obesity (MUO). We then divided the population into eight phenotypes based on obesity and the number of metabolic risk factors. Furthermore, the associations of eight phenotypes, based on obesity and specific metabolic risk factors, with depression were assessed.

**Result:**

Among all participants, a higher risk of depression was observed for MUNO, MHO and MUO than for MHNO. The risk was highest for MUO (OR = 1.442; 95% CI = 1.432, 1.451). However, the association between MHO and depression was different for men and women (OR = 0.941, men; OR = 1.132, women). The risk of depression increased as the number of metabolic risk factors increased. Dyslipidemia was the strongest metabolic risk factor. These relationships were consistent among patients ≥ 45 years of age.

**Conclusions:**

The increased risk of obesity-related depression appears to partly depend on metabolic health status. The results highlight the importance of a favorable metabolic status, and even nonobese populations should be screened for metabolic disorders.

## Introduction

Depression is the most common neuropsychiatric disorder, affecting more than 20% of the population in the U.S (1). Studies have shown that depression is associated with an increased risk of suicide and increased costs of health care (2, 3). Multiple risk factors, such as gender, family history, psychological stress, and alcohol, have been found to be associated with the occurrence of depression (4, 5). As a modifiable risk factor, obesity is considered to be a risk factor for depression (6–8); however, the results remain controversial. A few studies have identified a relationship between obesity and depression only among women (9–11), while others have identified no relationship between obesity and depression (12). In addition, Mounting evidence reveals that obesity-related metabolic dysfunction, including inflammation, insulin and leptin resistance, and hypertension, have emerged as key risks to depression (13). Metabolic dysfunction and inflammation are related to structural and functional changes in the brain and contribute to the development of depression (14, 15). Obesity-related metabolic abnormalities as an inflammatory disease may be an important neurobiological pathway affecting depression, rather than obesity *per se*.

Obesity is a serious health problem, affecting more than 10% of adults worldwide (16). Obese individuals have different metabolic characteristics and exhibit different metabolic obesity phenotypes (17, 18). Some obese individuals do not develop metabolic disorders (19, 20) and are described as having metabolically healthy obesity (MHO). In addition, many nonobese individuals with an abnormal metabolic health status are known to have metabolically unhealthy nonobesity (MUNO) (21–23). These two obesity phenotypes have attracted substantial attention recently, but only a few studies have described the relationship between obesity phenotypes and the risk of depression (24–26). An English longitudinal study (24)demonstrated that metabolically unhealthy obesity (MUO) was associated with the development of depression, although MHO was not. In contrast, a pooled analysis of eight studies showed that MHO was associated with depression and was not a healthy phenotype (25). Therefore, further research is needed to confirm these differences in the results. The combination of obesity and metabolic health status might be a better predictor of the development of depression than obesity alone, and this approach could enhance our knowledge of the composition of metabolic abnormalities and further reveal precise targets for treatment.

Evidence about the joint effects of obesity and different metabolic profiles on the risk of depression is limited. In addition, since depression and metabolic health status often present differently in different sex and age groups, whether this association is modified by sex and age is also worth investigating. Thus, we combined obesity and metabolism by focusing on metabolic obesity phenotypes and analyzed the Nationwide Inpatient Sample database to comprehensively investigate the relationship of obesity and metabolism with depression. Additionally, we analyzed sex-specific associations and the modifying role of age in this relationship.

## Materials and Methods

### Data Source

This was a cross-sectional study using data from 2016 to 2018 that were obtained from the Nationwide Inpatient Sample (NIS) database (http://hcup-us.ahrq.gov/nisoverview.jsp), a part of the Healthcare Cost and Utilization Project (HCUP). The NIS database is considered to be the largest all-payer database of hospital inpatients in the United States. The NIS collects inpatient health information from more than 1000 hospitals across a number of states, which account for approximately 20% of all inpatient admissions in the U.S. each year. The information extracted from this database included patient demographics and diagnostic procedural codes. The NIS is publicly available and contains no personal information. Therefore, institutional review board approval was not required for this study.

### Data Collection

The data were collected from the NIS database. Patients were identified according to the International Classification of Diseases (tenth revision) Clinical Modification (ICD-10-CM) procedural codes. We included all patients with the diagnosis of depression in our analysis. The patients who had depression included major depressive disorders and mood or dysthymic disorders with depressive features. The ICD-10-CM codes for patients who had depression were listed as F0631; F0632; F32.x- F33.x; F341. Information about the ICD-10-CM codes for obesity, major metabolic abnormalities, and other diagnoses is provided in **Supplementary Table 1**. Patients who were younger than 18 years of age, underweight or pregnant; had kidney failure, coagulation failure, respiratory failure, or circulatory failure; or died in the hospital were excluded.

### Definitions

The BMI ≥ 25 kg/m2, the WHO-defined cutoff for separating normal weight from overweight and obesity, was used to define obesity. Metabolic risk factors were defined as the following three components of metabolic syndrome based on the diagnostic criteria for metabolic syndrome (27, 28): (1) hypertension, (2) dyslipidemia (including hypertriglyceridemia and hypercholesterolemia), and (3) hyperglycemia (including prediabetes and diabetes).

Participants were categorized into four different phenotype groups based on their obesity and metabolic statuses: (1) metabolically healthy nonobesity (MHNO): patients were nonobese and had fewer than two metabolic risk factors; (2) MHO: patients were obese and had fewer than two metabolic risk factors; (3) MUNO: patients were nonobese and had at least two metabolic risk factors; and (4) MUO: patients were obese and had at least two metabolic risk factors.

In addition, two other groups of patients with metabolic obesity phenotypes were evaluated: patients with a particular number of metabolic risk factors and patients with specific metabolic risk factors. All the participants were classified into eight different phenotype groups according to obesity status and number of metabolic risk factors: (1) nonobese patients with no metabolic risk factors; (2) nonobese patients with one metabolic risk factor; (3) nonobese patients with two metabolic risk factors; (4) nonobese patients with three metabolic risk factors; (5) obese patients with no metabolic risk factors; (6) obese patients with one metabolic risk factor; (7) obese patients with two metabolic risk factors; and (8) obese patients with three metabolic risk factors.

Moreover, the participants were also classified into eight different phenotype groups according to obesity status and presence of specific metabolic risk factors: (1) nonobese patients with no metabolic risk factors; (2) nonobese patients with hyperglycemia; (3) nonobese patients with hypertension; (4) nonobese patients with dyslipidemia; (5) obese patients with no metabolic risk factors; (6) obese patients with hyperglycemia; (7) obese patients with hypertension; and (8) obese patients with dyslipidemia.

### Statistical Analysis

The baseline characteristics of patients with different obesity phenotypes are presented as the median (interquartile range) or proportion. Continuous variables were analyzed with the Kruskal–Wallis test, and the χ^2^ test was used to compare categorical variables across obesity phenotype groups. The associations between depression and different metabolic obesity phenotypes were investigated by logistic regression analysis. We further examined the effect of obesity combined with the number of metabolic risk factors or the presence of specific metabolic risk factors on depression. Subgroup analyses were performed based on the sex and age of the participants. In multivariate logistic models, we adjusted for sex, age, race, smoking, alcohol consumption, chronic kidney disease, chronic respiratory disease, liver-related diseases, HIV infection, and coronary heart disease. All the reported P values are two-sided. SPSS version 25 (SPSS, Chicago, IL, USA) was used for all the analyses.

## Results

### Characteristics of Participants According to Obesity Phenotype

A total of 9,022,089 participants (4148969 men and 4873120 women) were enrolled in this study. The characteristics of the participants with different obesity phenotypes are summarized in **Table 1**. The obesity prevalence was 16.4%, and 8.3% of participants had MHO and 28.1% of participants had MUNO. Compared with the metabolically healthy groups, the metabolically unhealthy groups had higher proportions of older individuals and smokers.

**Table 1:** Characteristics of the study population according to the different obesity phenotypes.

Participants (n)	Non-obese		Obese		P value*
	Metabolically healthy (MHNO)	Metabolically unhealthy (MUNO)	Metabolically healthy (MHO)	Metabolically unhealthy (MUO)	
	(n = 5,005,898)	(n = 2,534,735)	(n = 751,534)	(n = 729,922)	—
Age (years)	55 (31)	70 (19)	51 (23)	62 (17)	<0.001
Female (%)	53.5	50.7	65.7	57.0	<0.001
Smokers (%)	17.0	26.2	20.3	27.9	<0.001
Drinkers (%)	9.5	4.6	4.5	2.9	<0.001
Race (%)					<0.001
White	69.7	71.9	67.4	70.7	
Black	13.8	12.7	16.7	15.8	
Hispanic	10.6	9.4	11.6	9.5	
Asian/Pacific Islander	2.2	2.7	0.9	1.0	
Native Americans	0.6	0.5	0.6	0.6	
Others	3.1	2.8	2.7	2.3	
Metabolic risk factors (%)					<0.001
hypertension	30.6	95.7	39.7	94.9	
dyslipidemia	5.9	70.7	6.4	66.5	
hyperglycemia	5.2	53.4	9.2	69.0	

The data are summarized as the median (interquartile range) for continuous variables or as a numerical proportion for Categorical variables. *P values were for the Kruskal–Wallis test or the χ^2^ test across the four categories of obesity phenotypes.

**Figure 1:**
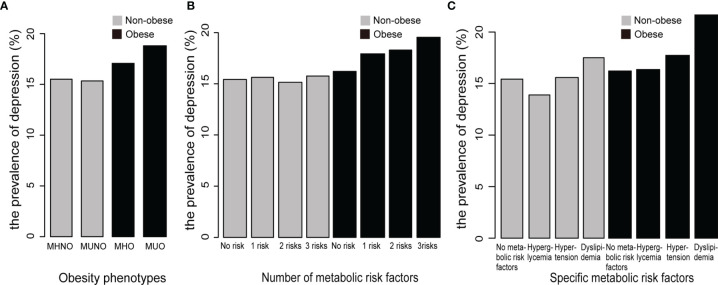
The prevalence of depression among the different metabolic obesity phenotypes. **(A)** The prevalence of depression among different obesity phenotypes. **(B)** The prevalence of depression among the phenotypes with different numbers of metabolic risk factors. **(C)** The prevalence of depression among the phenotypes with different specific metabolic risk factors. MHNO, metabolically healthy nonobesity; MUNO, metabolically unhealthy nonobesity; MHO, metabolically healthy obesity; MUO, metabolically unhealthy obesity.

In the MHNO, MUNO, MHO, and MUO phenotype groups, the prevalence of depression was 15.5%, 15.3%, 17.1%, and 18.8%, respectively (**Figure 1A**). Among the obese participants, the prevalence of depression increased as the number of metabolic risk factors increased (**Figure 1B**). In addition, the prevalence of depression was highest among participants with dyslipidemia regardless of whether these patients were obese or nonobese (**Figure 1C**).

### Associations of the Different Obesity Phenotypes With Depression

Overall, obese participants with a healthy metabolic status had a mildly higher risk of depression than nonobese participants, but the risk was significantly higher when participants had a poor metabolic status. Among all the participants, the risk of depression was higher in patients with the MUNO (OR = 1.291; 95% CI = 1.285, 1.297) and MHO (OR = 1.066; 95% CI = 1.059, 1.073) phenotypes than in patients with the MHNO phenotype. Patients with the MUO phenotype (OR = 1.442; 95% CI = 1.432, 1.451) had the highest risk of depression (**Table 2**). Among the male participants, the risk of depression was higher in the MUNO (OR = 1.265; 95% CI = 1.256, 1.275) and MUO (OR= 1.306; 95% CI = 1.291, 1.321) groups than in the MHNO group (**Table 2**). In addition, MHO seemed to be a protective factor for depression in men (OR = 0.941; 95% CI = 0.929, 0.953). However, among the female participants, compared to the MHNO group, the other three groups had a higher risk of depression (**Table 2**). The relationship between depression and obesity phenotype was significantly stronger for MUNO (OR = 1.263; CI = 1.257, 1.269), MHO (OR = 1.132; 95% CI = 1.124, 1.139) and MUO (OR = 1.458; 95% CI = 1.449, 1.468). The association between obesity phenotype and depression was consistent among patients ≥ 45 years of age (**Supplementary Figure 1**).

**Table 2:** Associations between depression and the different obesity phenotypes.

Variable	Total participants*	Men^#^	Women^#^
	OR (95% CI)	OR (95% CI)	OR (95% CI)
MHNO	Reference	Reference	Reference
MUNO	1.291 (1.285-1.297)	1.265 (1.256-1.275)	1.263 (1.257-1.269)
MHO	1.066 (1.059-1.073)	0.941 (0.929-0.953)	1.132 (1.124-1.139)
MUO	1.442 (1.432-1.451)	1.306 (1.291-1.321)	1.458 (1.449-1.468)

^*^The model was adjusted for sex, age, race, smoking, alcohol consumption, chronic kidney disease, chronic respiratory disease, liver-related diseases, HIV infection, and coronary heart disease. ^#^The model was adjusted for age, race, smoking, alcohol consumption, chronic kidney disease, chronic respiratory disease, liver-related diseases, HIV infection, and coronary heart disease. MHNO, metabolically healthy nonobesity; MUNO, metabolically unhealthy nonobesity; MHO, metabolically healthy obesity; MUO, metabolically unhealthy obesity.

### The Risk of Depression Increased With An Increasing Number of Metabolic Risk Factors

**Table 3** shows that the risk of depression increased as the number of metabolic risk factors increased, with ORs (95% CI) of 1.279 (1.272, 1.286) (nonobese patients with one metabolic syndrome risk factor), 1.425 (1.417, 1.434) (nonobese patients with two metabolic syndrome risk factors), 1.610 (1.597, 1.623) (nonobese patients with three metabolic syndrome risk factors), 1.347 (1.336, 1.359) (obese patients with one metabolic syndrome risk factor), 1.537 (1.524, 1.551) (obese patients with two metabolic syndrome risk factors) and 1.769 (1.750, 1.788) (obese patients with three metabolic syndrome risk factors). However, obesity with no metabolic risk factors seemed to be a protective factor among the male participants (OR = 0.858; 95% CI = 0.841, 0.876), but a risk factor among the female participants (OR = 1.086; 95% CI = 1.076, 1.097) (**Table 3**). The risk of depression also increased as the number of metabolic risk factors increased in all the age subgroups (**Supplementary Figure 2**).

**Table 3:** Associations between depression and the number of metabolic risk factors.

Variable	Total participants*	Men^#^	Women^#^
	OR (95% CI)	OR (95% CI)	OR (95% CI)
Non-obese, no risk	Reference	Reference	Reference
Non-obese, 1 risk	1.279 (1.272-1.286)	1.211 (1.201-1.221)	1.252 (1.245-1.258)
Non-obese, 2 risks	1.425 (1.417-1.434)	1.345 (1.332-1.358)	1.379 (1.371-1.387)
Non-obese, 3 risks	1.610 (1.597-1.623)	1.557 (1.537-1.576)	1.543 (1.530-1.555)
Obese, no risk	1.003 (0.993-1.013)	0.858 (0.841-0.876)	1.086 (1.076-1.097)
Obese, 1 risk	1.347 (1.336-1.359)	1.152 (1.134-1.170)	1.386 (1.374-1.397)
Obese, 2 risks	1.537 (1.524-1.551)	1.338 (1.318-1.358)	1.544 (1.530-1.557)
Obese, 3 risks	1.769 (1.750-1.788)	1.596 (1.568-1.625)	1.758 (1.739-1.777)

^*^The model was adjusted for sex, age, race, smoking, alcohol consumption, chronic kidney disease, chronic respiratory disease, liver-related diseases, HIV infection, and coronary heart disease. ^#^The model was adjusted for age, race, smoking, alcohol consumption, chronic kidney disease, chronic respiratory disease, liver-related diseases, HIV infection, and coronary heart disease.

### Dyslipidaemia Was The Strongest Metabolic Risk Factor

In the next analysis, we investigated the relationship between depression and specific metabolic risk factors (**Table 4**). Hypertension, dyslipidemia, and hyperglycemia were associated with depression regardless of obesity status, and the highest risk was associated with dyslipidemia (nonobese patients with dyslipidemia alone OR = 1.521; 95% CI = 1.505, 1.537; obese patients with dyslipidemia alone OR = 1.707; 95% CI = 1.670, 1.746). This result was consistent across all the age subgroups (**Supplementary Figure 3**) and female participants (**Table 4**). However, among the male participants, dyslipidemia remained the highest risk factor (nonobese patients with dyslipidemia alone OR = 1.361; 95% CI = 1.337, 1.385; obese patients with dyslipidemia alone OR = 1.360; 95% CI = 1.302, 1.420), but hyperglycemia was not associated with depression (nonobese patients and hyperglycemia alone OR = 0.996; 95% CI = 0.978, 1.014) (**Table 4**).

**Table 4:** Associations between depression and specific metabolic risk factors.

Variable	Total participants*	Men^#^	Women^#^
	OR (95% CI)	OR (95% CI)	OR (95% CI)
Non-obese, no risk	Reference	Reference	Reference
Non-obese, hyperglycemia	1.047 (1.035-1.059)	0.996 (0.978-1.014)	1.092 (1.075-1.108)
Non-obese, hypertension	1.284 (1.276-1.292)	1.252 (1.241-1.264)	1.303 (1.293-1.314)
Non-obese, dyslipidemia	1.521 (1.505-1.537)	1.361 (1.337-1.385)	1.635 (1.613-1.657)
Obese, no risk	1.014 (1.004-1.024)	0.865 (0.848-0.883)	1.092 (1.080-1.105)
Obese, hyperglycemia	1.144 (1.121-1.167)	0.958 (0.920-0.998)	1.241 (1.213-1.271)
Obese, hypertension	1.361 (1.348-1.375)	1.190 (1.169-1.212)	1.461 (1.444-1.478)
Obese, dyslipidemia	1.707 (1.670-1.746)	1.360 (1.302-1.420)	1.893 (1.844-1.944)

^*^The model was adjusted for sex, age, race, smoking, alcohol consumption, chronic kidney disease, chronic respiratory disease, liver-related diseases, HIV infection, and coronary heart disease. ^#^The model was adjusted for age, race, smoking, alcohol consumption, chronic kidney disease, chronic respiratory disease, liver-related diseases, HIV infection, and coronary heart disease.

## Discussion

The current study described the relationship between metabolic obesity phenotypes and the risk of depression based on data from the NIS database. Our study comprehensively analyzed the role of obesity and metabolic health status as risk factors for depression. Overall, we demonstrated that the MUO and MUNO phenotypes were independently related to a higher risk of depression, regardless of sex. Among women, the MHO phenotype was positively related to depression, whereas the opposite result was observed among men. In addition, the risk of depression among participants with dyslipidemia alone was significantly higher than that among participants with other metabolic risk factors.

In our study, we found that obesity is associated with depression, and metabolic abnormalities might have played a key role in this association. The bidirectional relationship between obesity and depression has been confirmed by many studies. Adipose expansion, gut microflora dysbiosis, peripheral immune activation and neuroinflammatory caused by obesity may contribute to the occurrence and development of depression (13). In addition, Metabolic dysfunction, including inflammation, insulin resistance, hypothalamic–pituitary–adrenal (HPA) axis dysregulation and central leptin resistance, have emerged as key risks to depression and anxiety development (7, 29, 30). Inflammatory responses to metabolic abnormalities such as dyslipidemia and hypertension also contribute to depression (31). Depression is associated with elevated circulating levels of proinflammatory cytokines, chemokines, and cell adhesion molecules, as well as prostaglandins (32, 33), and metabolic abnormalities are closely related to these inflammatory immunities (34). Thus, Metabolic abnormalities as an inflammatory disease may aggravate the effect of obesity on the neurobiological pathway of depression.

Studies have demonstrated sex-specific differences in the relationship between obesity and depression (35–38). Obesity might influence depressive symptoms through biological, psychosocial, and behavioral pathways (39, 40). However, those studies did not account for metabolic factors. Previous studies have investigated the relationship between the MHO phenotype and depression (8, 24, 25), although these studies did not account for sex and specific metabolic risk. Although a Korean study (26)showed that the association between MHO and depression was different among men and women, the evidence was insufficient. In our study, interestingly, obesity with no metabolic risk factor was found to be a protective factor among men. Although we found that the MHO phenotype was a protective factor among men, patients with the MHO phenotype might still have metabolic abnormalities. In the subsequent analysis, we found that nonobese individuals with specific metabolic risk factors were also at risk for depression. Therefore, the sex-specific difference in the MHO phenotype might be caused by simple obesity. Our results showed a sex-specific difference in the relationship between obesity and depression in the metabolically healthy population. This sex-specific difference may have caused the different results of previous studies, and our results in the current study fill an important gap in knowledge.

Although the biological mechanism underlying the sex-specific relationship between obesity and depression remains unknown, there are several possible explanations. One hypothesis is that obesity may impair progesterone and estrogen function and affect menstrual cycles and hormonal levels, which could influence the protective effect of female hormones on depression, mitochondrial function, and obesity (41). Furthermore, obesity is associated with different psychosocial profiles, and the association between obesity and depression is partly mediated by body-image satisfaction (42). Women might have greater stigmatization and social bias of obesity than men, which could lead to more severe depressive symptoms (43–45).

Another interesting result is that specific metabolic risk factors were significantly associated with depression in different age subgroups, with dyslipidemia having the strongest association with depression, although the components of dyslipidemia (e.g., high triglyceride levels, low high-density lipoprotein levels, or high cholesterol levels) were not analyzed. A bidirectional association between depression and MetS has been reported (46, 47). However, individual components of MetS are also important in driving this association. Multiple studies have demonstrated that hypertension is an independent risk factor for depression (48, 49). In addition, the association of depression with diabetes mellitus has been investigated in previous studies (50, 51), and a diagnosis of diabetes doubles the odds of comorbid depression. To our knowledge, no study has simultaneously compared the effects of hypertension, hyperglycemia, and dyslipidemia on depression. A study suggested that hyperlipidemia was significantly associated with depression and that statins could decrease the risk of depression in hyperlipidemic patients (52). Moreover, another study demonstrated that lipid levels were related to the development of depression and the severity of depression (53). Further research is needed to assess whether dyslipidemia is associated with a higher risk of depression than hyperglycemia or hypertension.

Immune inflammatory changes and oxidative stress have been associated with depression (54–57), and these factors may mediate the relationship between hyperlipidemia and depression. In addition, considering the fact that patients with depression generally live very unhealthy lives (58, 59), unhealthy lifestyles might be more likely to lead to obesity and dyslipidemia. Additional cohort studies are needed to further investigate the conclusions of this study.

This study had several unique strengths. First, the study included a large sample size, and therefore, the results are generalizable and representative. Second, we performed a comprehensive evaluation of the associations among obesity, metabolic risk factors, and depression. Finally, we performed a more detailed analysis to better understand obesity and individual components of metabolic syndrome in relation to depression. However, there were some limitations of our study. First, although our large study population enhanced the generalizability of our results, this was an observational study, and we were unable to infer causality in the findings. Second, potentially confounding variables, including antidepressant medication use, family history, and socioeconomic status, were not provided in the NIS database. Furthermore, the connection between obesity, metabolic abnormalities and depression can’t exclude the analysis of eating behaviors. Finally, the study could not address the extent to which metabolic abnormalities represent adverse metabolic effects of psychotropic drugs.

In conclusion, the present results indicated that MUO and MUNO were associated with a higher risk of depression than MHNO. Furthermore, among female participants, metabolically healthy obesity was related to a higher risk of depression. The risk of depression increased as the number of metabolic risk factors increased. In addition, dyslipidemia had the strongest association with depression. Our findings highlight the importance of both metabolic health status and obesity in depression and suggest a compelling need to improve metabolic health status regardless of the presence of obesity. Furthermore, our results might provide a basis for further studies to investigate individualized MetS-related treatment strategies for depression, and this hypothesis requires further examination.

## Data Availability Statement

The original contributions presented in the study are included in the article/**Supplementary Material**. Further inquiries can be directed to the corresponding authors.

## Ethics Statement

Ethical review and approval was not required for the study on human participants in accordance with the local legislation and institutional requirements. Written informed consent for participation was not required for this study in accordance with the national legislation and the institutional requirements.

## Author Contributions

ZW, YL, and LG designed the study and wrote the manuscript. ZY, TB, and YW provided oversight to the study analyses. YC, FZ, and QL are responsible for data cleansing. XF and JH revised the manuscript and had final approval of the submitted and published versions. LG is the guarantor of this work and, as such, had full access to all the data in the study and take responsibility for the integrity of the data and the accuracy of the data analysis. All authors contributed to the article and approved the submitted version.

## Funding

This work was supported by National Key Research and Development Program of China (2021YFA0805100 and 2017YFC1309800).

## Conflict of Interest

The authors declare that the research was conducted in the absence of any commercial or financial relationships that could be construed as a potential conflict of interest.

## Publisher’s Note

All claims expressed in this article are solely those of the authors and do not necessarily represent those of their affiliated organizations, or those of the publisher, the editors and the reviewers. Any product that may be evaluated in this article, or claim that may be made by its manufacturer, is not guaranteed or endorsed by the publisher.
