# Supplementary material for: The Relationship Between Obesity and Depression Is Partly Dependent on Metabolic Health Status: A Nationwide Inpatient Sample Database Study
Source: Front Endocrinol (Lausanne). 2022 May 25;13:880230. doi: 10.3389/fendo.2022.880230 (PMC9174461; doi:10.3389/fendo.2022.880230)
Supplement: Supplementary file 1 [file DataSheet_1.docx]

**Supplementary Table 1. ICD-10-CM codes for disease**

| **Variables** | **ICD-10-CM diagnostic codes** |
| --- | --- |
| Alcohol consumption | F1010- F1099 |
| Chronic kidney disease | N181; N182; N183; N1830; N1831; N1832; N184; N185; N186; N189; |
| Chronic respiratory disease | J40; J410; J411; J418; J430; J431; J432; J438; J439; J440; J441;J449; J4520; J4521;J4522;J4530; J4531; J4532; J4540; J4541; J4542; J4550; J4551; J4552; J45901; J45902 J45909; J4990; J45991; J45998; J470; J471; J479; |
| Circulatory failure | O2912.x; I0981; I110; I130; I50.x |
| Coagulation failure | D65- D68 |
| Coronary heart disease | I2101- I25812 |
| Depressive disorder | F0631; F0632; F32.x- F33.x; F341 |
| Dyslipidaemia | E782; E784; E7849; E785 |
| HIV infection | B20, B9735, Z21 |
| Hyperglycaemia | R7303; E0800-E089; E1010-E119 |
| Hypertension | I10.x; I110; I119; I120; I129; I130; I1310; I1311; I132; I150; I151; I152; I158; I159 |
| Kidney failure | N170- N172, N178, N179, N19, N990, R34, R944, I120, I1310, I1311, N181- N186, N189, N19, N250, Z4901, Z4902, Z940 |
| Liver-related diseases | K70-K77, Z944, I864, B15- B19, I85(I850- I8511) |
| Obesity | Z6825 - Z6845; E663; E669; E6601; E662 |
| Pregnancy | Z33.x-Z34.x; Z36.x; Z3A.x; O000- O002; O008- O009; O09.x- O12.x; |
| Respiratory failure | J80; J960.x |
| Smoking | F17200- F17299; Z716; Z720; T65211A-T65294S |
| Underweight | R636; Z681 |

**Supplementary figure 1. The association of obesity phenotypes with depression in the different age subgroups**


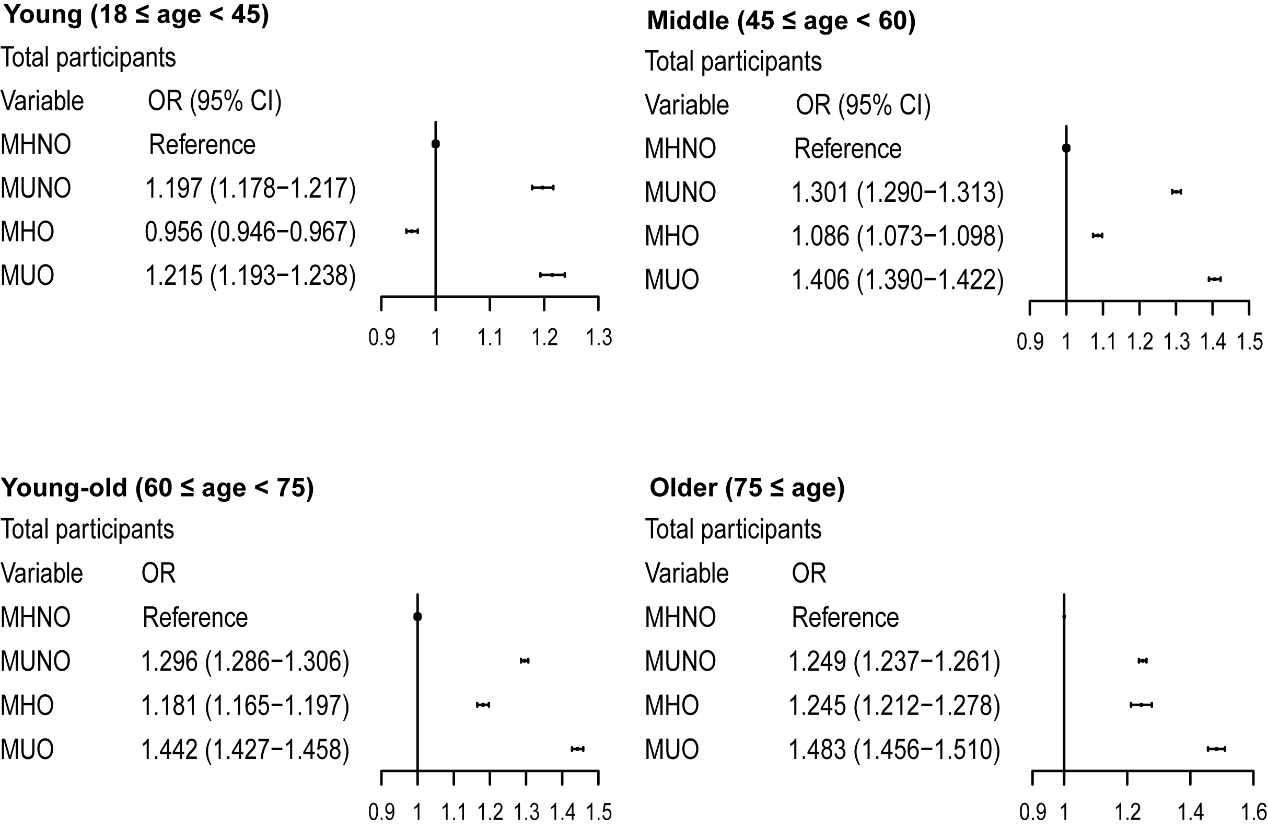


MHNO: metabolically healthy nonobesity, MUNO: metabolically unhealthy nonobesity, MHO: metabolically healthy obesity, MUO: metabolically unhealthy obesity. The model was adjusted for sex, race, smoking, alcohol consumption, chronic kidney disease, chronic respiratory disease, liver-related diseases, HIV infection, and coronary heart disease.

**Supplementary figure 2.** **The association of obesity and the number of metabolic risk factors with depression in the different age subgroups**


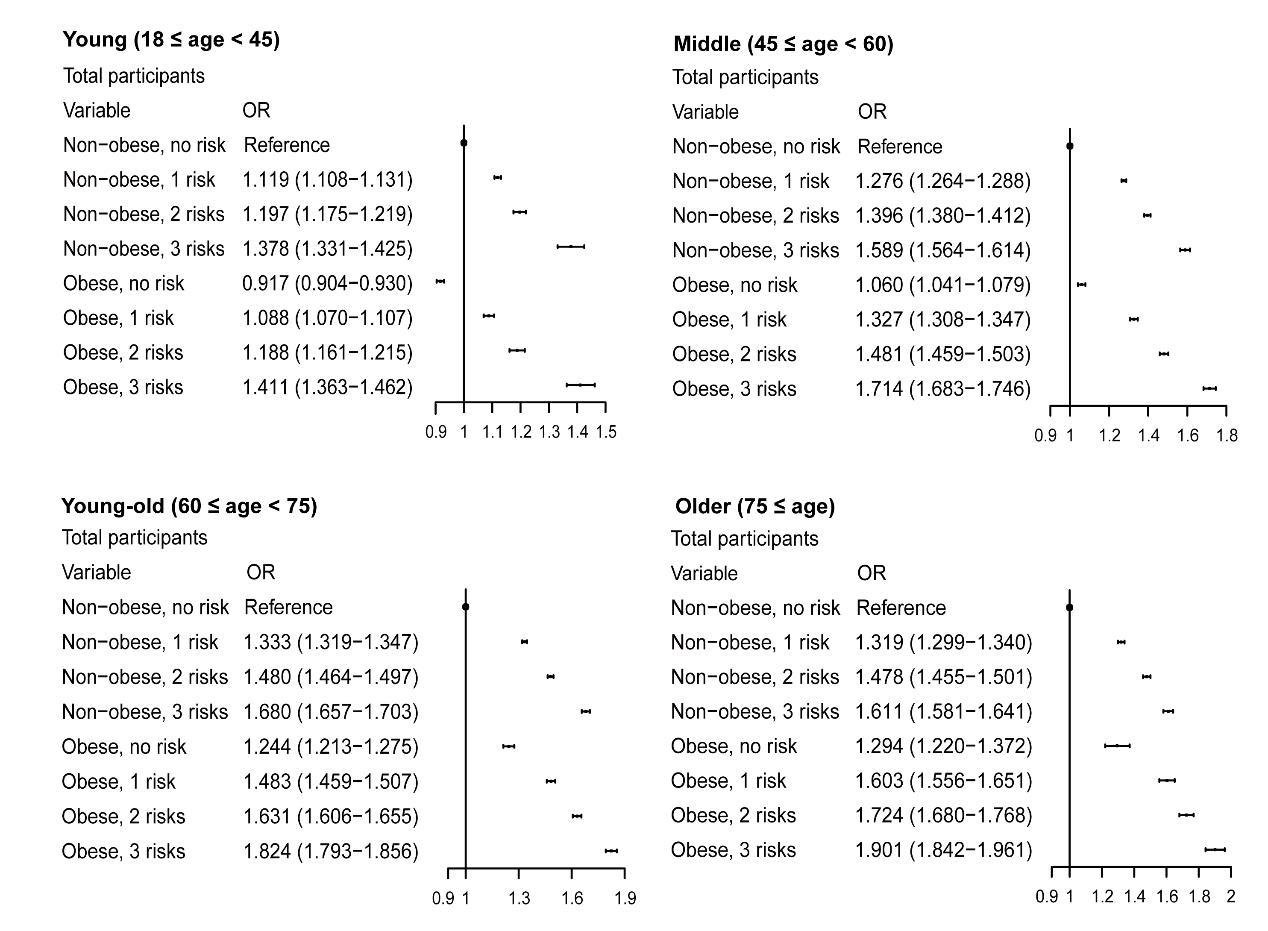
The model was adjusted for sex, race, smoking, alcohol consumption, chronic kidney disease, chronic respiratory disease, liver-related diseases, HIV infection, and coronary heart disease.

**Supplementary figure 3. The association of obesity and specific metabolic risk factors with depression in the different age subgroups**


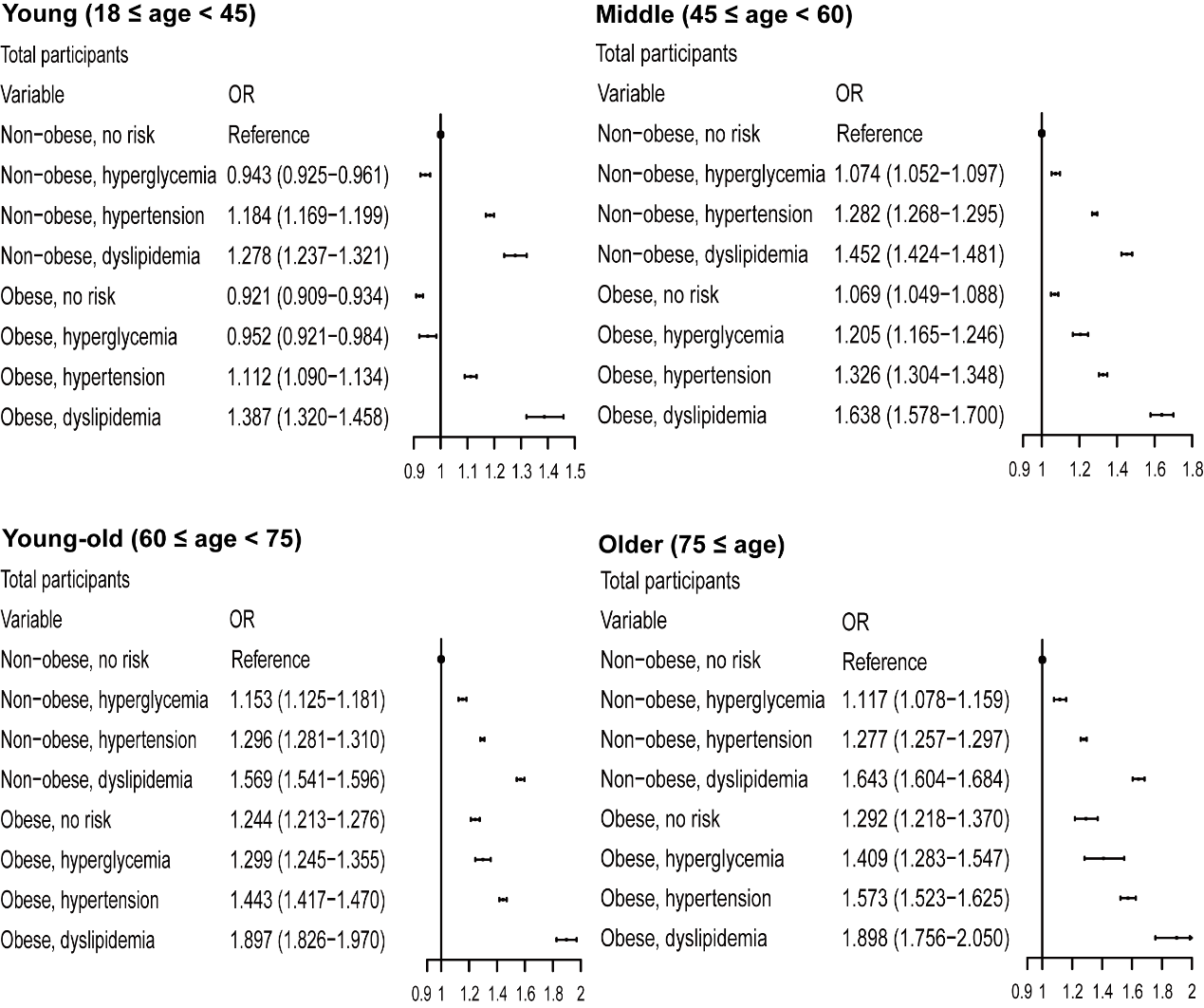
The model was adjusted for sex, race, smoking, alcohol consumption, chronic kidney disease, chronic respiratory disease, liver-related diseases, HIV infection, and coronary heart disease.
